# Evaluation of the Effect of Gene Duplication by Genome Editing on Drug Resistance in *Plasmodium falciparum*

**DOI:** 10.3389/fcimb.2022.915656

**Published:** 2022-07-05

**Authors:** Rie Kubota, Tomoko Ishino, Shiroh Iwanaga, Naoaki Shinzawa

**Affiliations:** ^1^ Department of Parasitology and Tropical Medicine, Graduate School of Medical and Dental Sciences, Tokyo Medical and Dental University, Tokyo, Japan; ^2^ Department of Molecular Protozoology, Research Institute for Microbial Diseases, Osaka University, Osaka, Japan

**Keywords:** *Plasmodium falciparum*, CRISPR/Cas9, *mdr1*, plasmepsin, drug resistance

## Abstract

The emergence and spread of drug-resistant *Plasmodium falciparum* have compromised antimalarial efficacy and threatened the global malaria elimination campaign using artemisinin combination therapies. The impacts of amino acid substitutions in antimalarial drug resistance-associated genes on drug susceptibility have been investigated; however, the effects of amplification of those genes remain unexplored due to the lack of robust genetic approaches. Here, we generated transgenic *P. falciparum* parasites with an additional copy of a drug resistance-associated gene using the highly efficient CRISPR/Cas9 system and investigated their drug response. Insertion of a drug resistance-associated gene expression cassette in the genome resulted in a roughly twofold increase of mRNA levels of the target gene *mdr1*, which encodes multidrug resistance protein 1. The gene duplication event contributed to resistance to mefloquine, lumefantrine, and dihydroartemisinin, while the duplication of a genomic region encoding plasmepsin 2 and plasmepsin 3 did not affect resistance to antimalarial drugs, including piperaquine. We further demonstrated that *mdr1* mRNA expression levels are strongly associated with mefloquine resistance in several field-derived *P. falciparum* lines with various genetic backgrounds. This study provides compelling evidence that *mdr1* could serve as a molecular marker for the surveillance of mefloquine-resistant parasites. Long DNA integration into parasite genomes using the CRISPR/Cas9 system provides a useful tool for the evaluation of the effect of copy number variation on drug response.

## Introduction

The WHO estimated that there were 241 million cases and 627,000 deaths due to malaria in 2020 (World Malaria Report, 2021). The emergence of drug-resistant parasites makes malaria control difficult, as evidenced by the fact that chloroquine-resistant *Plasmodium falciparum* is now spread globally (Ecker et al., 2012). Currently, the WHO recommends artemisinin-based combination therapies (ACTs), in which artemisinin is used as a first-line drug along with partner drugs such as mefloquine, lumefantrine, piperaquine, and amodiaquine. ACTs have greatly contributed to the decrease in malarial deaths (Bhatt et al., 2015; World Malaria Report, 2021). However, recent epidemiological studies indicate that ACT treatment failures are increasing worldwide, particularly in the Greater Mekong Subregion (GMS) (Ashley et al., 2014; Amato et al., 2017; Witkowski et al., 2017; Ikeda et al., 2018). One possible reason for ACT failures is the emergence of strains with mutations in *kelch13*, which are associated with artemisinin resistance (Ariey et al., 2014; van der Pluijm et al., 2019; Balikagala et al., 2021). In addition, ACT failure may result from resistance acquired from partner drugs (Leang et al., 2013; Ashley et al., 2014; Nsanzabana, 2019). Triple ACTs (TACTs), which add another partner drug with a conventional ACT, were introduced and have increased antimalarial efficacy (Dini et al., 2018; Okell et al., 2018; van der Pluijm et al., 2020). The selection of effective partner drugs is important for successful treatment by ACTs; the identification of suitable molecular markers associated with partner drug resistance is required for optimal ACT design and implementation.

Recent epidemiological studies suggested that partner drug resistance is due to amino acid substitutions or copy number variations of several genes. Mefloquine resistance is considered to be conferred by an increase in the copy number of the gene that encodes multidrug resistance protein 1 (PF3D7_0523000, *mdr1*), a protein homologous to the human drug efflux pump (Price et al., 2004). Piperaquine resistance is reported to be correlated with increased copy number of plasmepsin 2 (PF3D7_148000, *pm2*) and plasmepsin 3 (PF3D7_148100, *pm3*), based on genome-wide association studies (Amato et al., 2017; Witkowski et al., 2017). The relationship between specific amino acid substitutions and drug resistance has been analyzed by allelic replacement using plasmid integration *via* a single crossover, as well as recently developed genome editing methods such as zinc-finger nuclease or CRISPR/Cas9 (Triglia et al., 1998; Sidhu et al., 2005; Veiga et al., 2016; Dhingra et al., 2017; Ross et al., 2018). In contrast, the association between gene copy number variations and drug sensitivity has been not well examined due to the technical limitations of genetic methods (Sidhu et al., 2006; Loesbanluechai et al., 2019).

We recently utilized the CRISPR/Cas9 genome editing system to integrate a large gene expression cassette (Nishi et al., 2021). Here, we applied this technology to generate transgenic parasites in which an additional copy of *mdr1*, *pm2*, or *pm3* was integrated to examine the effect of their amplification on antimalarial drug sensitivities. Our results demonstrated that increased mRNA expression of *mdr1* reduced the sensitivity to mefloquine and slightly to lumefantrine and dihydroartemisinin. In contrast, duplication of the genomic region containing *pm2* and *pm3* did not change the sensitivity to known partner drugs, including piperaquine. We further demonstrated that *mdr1* expression levels are associated with mefloquine resistance using field-derived strains, and we illustrated that *mdr1* expression and/or copy number could be a useful marker for the surveillance of mefloquine-resistant parasites. This is the first report of the generation of transgenic parasites with an additional copy of a drug resistance-associated gene. Our robust genome editing protocol to introduce gene amplification could be an effective tool to evaluate the association between copy number variation and drug resistance.

## Materials and Method

### Ethical Clearance

Ethical approval for the use of human red blood cells (RBCs) and plasma from the Japanese Red Cross Tokyo Blood Center was obtained from the Medical Research Ethical Committee of the Tokyo Medical and Dental University.

### Parasite Strains and Culture

All laboratory strains of *P. falciparum* were obtained from the MR4 repository (www.beiresources.org), namely, 3D7 (MRA-102), Dd2 (MRA-150), IPC_5188 (MRA-1239), IPC_5202 (MRA-1240), 7G8 (MRA-125), and FCR3 (MRA-736). The pfcas9 parasite line was used for the derivation of the transgenic parasites in this study. This line was generated from the *P. falciparum* strain 3D7 in our previous study (Nishi et al., 2021) and possesses a cas9 expression cassette integrated within the knob-associated histidine-rich protein locus (PF3D7_0202000, *kahrp*). All parasite strains were cultivated with human type O RBCs (obtained from the Japanese Red Cross Tokyo Blood Center) at 2% hematocrit in a complete medium, which consists of RPMI-1640 medium containing 2.5% human serum (obtained from the Japanese Red Cross Tokyo Blood Center), 2.5% AlbuMAX II (Life Technologies, Carlsbad, CA, USA), 25 mM of HEPES, 0.225% sodium bicarbonate, and 0.38 mM of hypoxanthine supplemented with 10 μg/ml gentamicin. Parasite cultures were incubated under low-oxygen conditions (90% N_2_, 5% CO_2_, and 5% O_2_) as described (Nishi et al., 2021).

### Construction of an SgRNA-Expressing Plasmid and Preparation of Donor Template DNA

SgRNA-expressing plasmids and donor template DNA were generated as described below and used for the derivation of transgenic parasites with an additional copy of *mdr1*, *pm2*, *pm3*, or both *pm2* and *pm3*, respectively named pfcas9-mdr1, pfcas9-pm2, pfcas9-pm3, and pfcas9-pm2/3. The gRNA was designed as described (Nishi et al., 2021). Briefly, a 19-bp gRNA target sequence, which is predicted to have no off-target candidates, was designed using the CHOPCHOP program (https://chopchop.cbu.uib.no/). To generate the plasmid expressing sgRNA, an oligonucleotide pair was annealed and cloned into the pf-gRNA plasmid as described. The plasmid expressing sgRNA was modified by a csp-sgRNA_1F and csp-sgRNA_1R primer set to integrate within the circumsporozoite protein locus (*csp*, PF3D7_0304600) and named psgRNA-csp. To integrate the *mdr1* promoter region within the *csp* locus, the plasmid expressing sgRNA to recognize csp-HR1 was generated with the csp-gRNA_2F and csp-sgRNA_2R primer set, and the resulting plasmid was named psgRNA-cspHR1. To integrate the *pm3* region within the *csp* locus of pfcas9-pm2, the plasmid expressing sgRNA to recognize csp-HR2 was generated with the csp-gRNA_3F and csp-sgRNA_3R primer set, and the resulting plasmid was named psgRNA-cspHR2. The donor DNA plasmid pDonor_mdr1 and PCR-fragment were used for the generation of the pfcas9-mdr1 parasite. First, the *mdr1* fragment including ORF and 3′ untranslated regions (UTRs) was inserted using an In-Fusion HD cloning kit into the EcoRI and HindIII sites of the donor template DNA plasmid, to replace the GFP coding sequence. Next, to add the 3-kbp promoter of *mdr1* to the *csp* locus, the donor DNA fragment was used in which the homologous region of the *csp* locus and *mdr1* region containing the promoter and 700-bp ORF was fused by overlap PCR. To prevent re-cleavage by the Cas9–sgRNA complex, the donor DNA lacked 20 bp of the *csp* sequence in the homologous region. The donor DNA plasmids pDonor_pm2 and pDonor_pm3 were used for the generation of pfcas9-pm2 or pfcas9-pm3 parasites, respectively. The donor DNA plasmids were modified by integrating the *pm2* fragment including promoter and 3′-UTR and amplified using the primer set pm2_HindIII-F and pm2_EcoRI-R, or the *pm3* fragment amplified using the primer set pm3_HindIII-F and pm3_EcoRI-R as described above. The donor DNA to generate the pfcas9-pm2/3 parasite was a fragment that fused the 3′-UTR of *pm2* with the *pm3* expression cassette by overlap PCR. All donor DNA plasmids were linearized for transfection. The sequences of oligonucleotides used for sgRNA-expressing plasmid or donor DNA constructions are listed in **Supplementary Table 1**.

### Generation of Transgenic Parasites

Transfection of *P. falciparum* by CRISPR/Cas9 was performed as described (Mohring et al., 2019; Morita et al., 2021). Tightly synchronized mature schizonts were transfected using the FP-158 program on a Nucleofector 4D device (LONZA, Basel, Switzerland). First, for transfections to integrate the target gene into the *csp* locus, the psgRNA-csp and linear donor DNA were used. Next, the psgRNA-cspHR1 and linear donor DNA were used to add the *mdr1* promoter region. To generate the pfcas9-pm2/3 line, the pfcas9-pm2 line was transfected with linear donor DNA and the psgRNA-cspHR2. Each linear repair DNA template (25 µg) plus plasmid containing the sgRNA (25 µg) was dissolved in 100 µl of P3 Primary Cell 4D-Nucleofector™ X Kit (LONZA) and were mixed with purified schizonts (1 × 10^8^). Pyrimethamine-mediated (25 ng/ml) selection of the transgenic parasites was initiated 72 h after transfection and continued for 10 days. The emergence of transgenic parasites was monitored using a diagnostic PCR assay, followed by 1 µM of 5-fluorocytosine treatment for 5 days to eliminate plasmid-containing parasites. Parasite clones were further selected by limiting dilution, in which by diagnostics the target gene was integrated. The diagnostic PCR products were sequenced by standard Sanger sequencing to confirm the absence of undesired mutations in the target gene coding regions. To generate pfcas9-mdr1 and pfcas9-pm2-3, synchronized mature schizonts of the plasmid-free primary transgenic parasite clone were used for the secondary transfection. Removal of the sgRNA plasmid from the primary transgenic parasites was confirmed by the restoration of pyrimethamine susceptibility. The sequences of primers used for diagnostic PCR are listed in **Supplementary Table 1**.

### Transcription of Target Genes

To quantify *mdr1* transcript levels, total RNA was purified from parasites 6 to 10 h post-infection (hpi), when the expression of *mdr1* reached the maximum level (https://plasmodb.org/plasmo). For assay of *pm2* and *pm3* transcript levels, total RNAs were purified from parasites 26 to 30 hpi. Total RNA was isolated using TRIzol™ (Thermo Fisher Scientific, Waltham, MA, USA) and purified from three biological independent samples for each parasite strain. The cDNAs were synthesized from 1 µg of each purified total RNA using a PrimeScript RT reagent kit with a gDNA Eraser according to the manufacturer’s instructions (TAKARA Bio, Kusatsu, Japan). Quantitative reverse transcription PCR assays were performed in triplicate using cDNA as a template with Power SYBR^®^ Green Master Mix (Thermo Fisher Scientific) and a StepOne Plus device (Thermo Fisher Scientific). Target gene expression levels were calculated based on the CT values using the ddCt method. All real-time PCRs were performed using the *seryl tRNA synthetase* gene (PF3D7_0717700) as an internal control and in at least two independent experiments. Primers are listed in **Supplementary Table 1**.

### Southern Blotting

Southern hybridization analysis was performed as described with some modifications (Shinzawa et al., 2020; Nishi et al., 2021). Briefly, to detect the *mdr1* locus, genomic DNA was extracted from blood-stage parasites (2 µg) and digested with the restriction enzyme ScaI. The primers mdr1-probe-F and mdr1-probe-R were used to generate the DNA probe. To detect the *pm2* locus, parasite genomic DNA was digested with the restriction enzymes ScaI and SalI. For detecting the *pm3* locus, parasite genomic DNA was digested with the restriction enzymes ScaI, SalI, and BamHI. The fragments were separated on a 0.8% agarose gel and transferred to nylon membranes. DNA probes to characterize the *pm2* and *pm3* loci were generated with the following respective primer pairs: pm2-probe-F and pm2-probe-R, and pm3-probe-F and pm3-probe-R. The positive control was the plasmid used to generate the transgenic parasites in this study. The PCR products were labeled with digoxigenin (DIG) and used as hybridization probes. Chemiluminescence signals were detected using ChemiDoc MP (Bio-Rad, Hercules, CA, USA).

### Fitness Assay

Fitness assays were performed as described with some modifications (Nishi et al., 2021). Tightly sorbitol-synchronized ring-stage parasites were inoculated at 0.1% parasitemia and 2% hematocrit in a drug-free complete medium. The culture medium was changed, and the parasitemia was monitored by Giemsa stain daily for 4 days.

### Preparation of Antimalarial Drug Stock Solutions

Mefloquine, piperaquine, lumefantrine, and dihydroartemisinin were purchased from Tokyo Chemical Industry Co., Ltd. Tokyo, Japan. Stock solutions of 50 mM of mefloquine, 2 mM of lumefantrine, and 100 mM of dihydroartemisinin were prepared in dimethyl sulfoxide (Wako, Tokyo, Japan) and 1 mM of piperaquine in 0.5% lactic acid (Wako), with ultrasonic treatment and warming at 60°C to dissolve. The stock solutions were aliquoted and stored at −20°C until use.

### Measurement of the IC_50_ Values of Antimalarial Drugs

Parasites were synchronized by the sorbitol-based method, and 48 h later, the parasitemia was adjusted to 0.5% in blood at 2% hematocrit. The final drug concentrations tested ranged from 0 to 800 nM for mefloquine, 0 to 100 nM for piperaquine, 0 to 1,600 nM for lumefantrine, and 0 to 80 nM for dihydroartemisinin. Parasite culture (50 µl) and diluted drug medium (50 µl) were dispensed at a final parasitemia of 0.25% in quadruplicate to wells of a flat-bottom 96-well plate. Piperaquine and lumefantrine were incubated for 96 h, mefloquine for 144 h due to its longer half-life, and dihydroartemisinin for 48 h due to its rapid loss of drug activity. Parasite growth in each well was assessed using SYBR Green I (Invitrogen, Waltham, MA, USA) with lysis buffer (20 mM of Tris-HCl, pH 7.5, 5 mM EDTA, 0.008% saponin, and 0.08% Triton X-100). The plates were shaken until all RBCs were lysed and were then placed at room temperature in the dark for 30 min. SYBR Green I fluorescence was measured using a BioTek Synergy LX microplate reader (Agilent Technologies, Santa Clara, CA, USA) with excitation and emission wavelengths at 485 and 535 nm, respectively. The IC_50_ values were calculated based on the detected fluorescence intensities using GraphPad Prism 9 software. Briefly, their survival rates were plotted against the logarithm of drug concentration, and the curve fittings were performed by non-linear regression to yield the drug concentration (IC_50_ values) that produced a 50% survival rate.

### Piperaquine Survival Assay

*In vitro* piperaquine survival assays (PSAs) were performed as described (Duru et al., 2015). Briefly, early ring-stage parasites at 0 to 3 hpi were prepared using 70% Percoll gradients (GE Healthcare, Chicago, IL, USA) and 5% sorbitol treatment. The resulting tightly synchronized parasites with 0.5% parasitemia and 2% hematocrit were cultured in 200 nM of piperaquine or 0.5% lactic acid medium for 48 h in 48-well plates. The parasites were washed with an incomplete medium and further cultured for 24 h without drug treatment. Parasitemias were determined *via* SYBR Green I fluorescence, as described above for the *in vitro* IC_50_ drug susceptibility assays. Good concordance was observed between cell count values by a SYBR Green assay versus microscopy-based counting of Giemsa-stained thin blood smears. Assays were repeated in duplicate at least twice.

### Copy Number Variation Analysis of *mdr1*

To quantify *mdr1* copy numbers, total genomic DNAs were purified from the transgenic parasites as described above. Real-time genomic PCR assays were performed in triplicate with Power SYBR^®^ Green Master Mix (Thermo Fisher Scientific) and a StepOne Plus device (Thermo Fisher Scientific) using genomic DNA as a template. To calculate the *mdr1* copy number, PF3D7_0523800 was used as an internal control. The primer sets for *mdr1* and PF3D7_0523800 were validated using pfcas9 and pfcasp-mdr1 genomic DNA. For laboratory strains, the reactions of real-time genomic PCR were performed using 0.1 ng of genomic DNA. Quantification of *mdr1* copy numbers was determined by ddCt methods. All real-time PCRs were performed in two independent experiments. Primers are listed in **Supplementary Table 1**.

### Statistical Analysis

All experiments were statistically analyzed from at least three biological replicates using an unpaired Student’s t-test within GraphPad Prism 9.0 (GraphPad Software Inc.).

## Results

### Generation of Transgenic *Plasmodium falciparum* Parasites With an Additional Copy of Drug Resistance-Associated Genes

**Figure 1:**
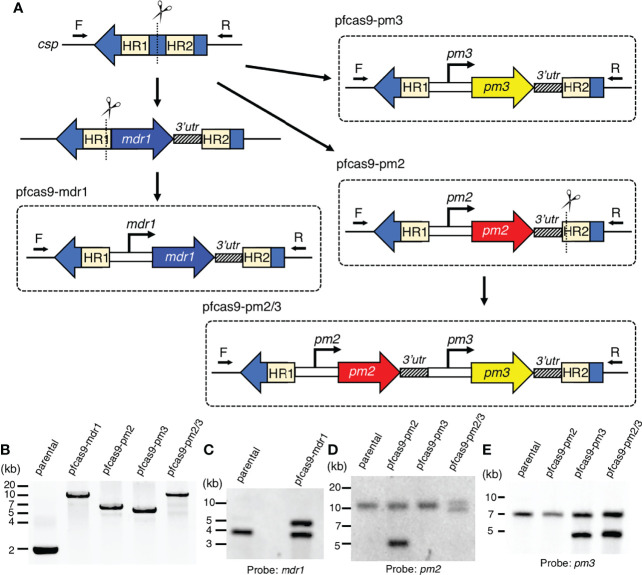
Generation of transgenic parasites with an additional copy of drug resistance-associated genes by CRISPR/Cas9-based genome editing. **(A)** Schematic representation of a strategy to derive transgenic parasites with an additional copy of drug resistance-associated genes. The pfcas9 parasite line was transfected with an sgRNA expression vector and a linear form of the donor DNA template. The drug resistance-associated gene expression cassette was integrated into the *csp* locus *via* homology-directed repair. **(B)** Genotyping PCR was performed using csp-check-F and csp-check-R primers. For each transgenic parasite, the integration of an additional copy into the *csp* locus was confirmed. **(C–E)** Southern hybridization analyses of transgenic parasites were carried out using partial DNA fragments of *mdr1* **(C)**, *pm2* **(D)**, and *pm3* **(E)** open reading frames as the DNA probes. A double signal indicating gene amplification was detected in each transgenic parasite.

To investigate a possible direct causal association of drug resistance with copy number amplification of *mdr1*, *pm2*, and *pm3*, we generated transgenic *P. falciparum* parasites with an additional copy of each gene by CRISPR/Cas9-mediated knocking-in of long genomic regions. The introduced expression cassettes contained the target gene coding regions together with the promoter and 3′-UTRs, predicted by amplification-free RNA-seq data to mimic its original expression pattern (Chappell et al., 2020). The above resistance-associated genes were introduced into the circumsporozoite protein (*csp*) locus of a *Streptococcus pyogenes* Cas9-expressing 3D7 parental strain (pfcas9; parental). The *csp* locus is considered to be dispensable for asexual parasite proliferation, with no known knock-out phenotype (Nishi et al., 2021). To knock-in *mdr1*, the fragment corresponding to the coding region and 3′-UTR was introduced into the *csp* locus, followed by the insertion of an upstream 3-kb promoter region (pfcas9-mdr1; **Figure 1A**). For *pm2* and *pm3*, transgenic parasites were generated with an additional copy of *pm2* or *pm3* (pfcas9-pm2 or pfcas9-pm3; **Figure 1A**). To investigate a possible synergistic effect of *pm2* and *pm3*, an expression cassette of *pm3* was introduced into the genome of pfcas9-pm2 downstream of the inserted *pm2*, to generate transgenic parasites containing additional copies of both *pm2* and *pm3* (pfcas9-pm2/3; **Figure 1A**). Genotyping PCR and Southern hybridization analyses showed the correct integration of the additional gene copy at the *csp* locus in the respective transgenic parasites (**Figures 1B–E**). There were no undesired mutations in the coding regions of the inserted genes (data not shown), supporting that the additional copies were correctly introduced.

### Increase in mRNA Expression of Target Genes Following Copy Number Amplification

**Figure 2:**
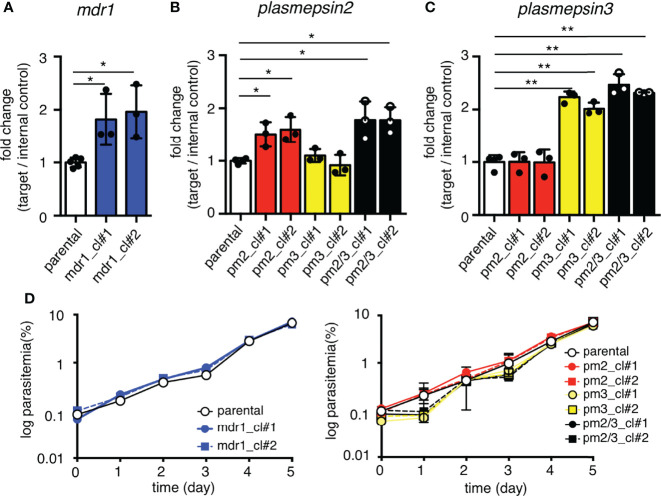
Increased mRNA expression by an additional copy of drug resistance-associated genes. **(A)** The mRNA expression of *mdr1* was measured by quantitative reverse transcription PCR. The two clones of transgenic parasites with integrated *mdr1* cassette (blue) had approximately 2-fold increased mRNA expression compared to the parental strain (white). pfcas9-mdr1 clones are labeled mdr1_cl#1 and mdr1_cl#2. **(B, C)** The mRNA expressions of *pm2* or *pm3* were measured by quantitative reverse transcription PCR. The *pm2*, *pm3*, or both-integrated parasites (red, yellow, and black) had approximately 2-fold increased mRNA expression of integrated genes compared to the parental strain. pfcas9-pm2, pfcas9-pm3, and pfcas9-pm2/3 clones are labeled pm2_cl#1 and pm2_cl#2, pm3_cl#1 and pm3_cl#2, and pm2/3_cl#1 and pm2/3_cl#2, respectively. **(D)** Growth of the transgenic parasites during asexual developmental cycle was comparable to the parental strain (left, *mdr1*; right, *pm2*, *pm3*, or both). N, n = 3–5 (**Supplementary Table 2**). Significance was determined using Student’s t-tests. *p < 0.01; **p < 0.001.

To evaluate the relationship between copy number amplification and mRNA expression, the transcript levels of each gene were compared between parental and transgenic parasites. The *mdr1* transcript levels were examined by quantitative reverse transcription PCR at the ring stage (6–10 hpi), and those of *pm2* and *pm3* were examined at the trophozoite stage (26–30 hpi), at their highest transcription timing according to the transcriptomics data (Toenhake et al., 2018; Wichers et al., 2019; Chappell et al., 2020). The results demonstrated that the *mdr1* transcript level in ring-stage parasites of the pfcas9-mdr1 line was about 2-fold higher than that of parental parasites (**Figure 2A** and **Supplementary Table 2**). The insertion of each *pm2* or *pm3* expression cassette, or both *pm2* and *pm3* increased their target gene mRNA levels by 1.5- to 2-fold compared to parental parasites (**Figures 2B, C** and **Supplementary Table 2**). All transgenic strains appeared to be normal with respect to intra-erythrocytic development (**Figure 2D**). The results were consistent for two independent clones of each transgenic parasite. In summary, these results demonstrated that an additional copy of a drug resistance-associated gene within the *csp* locus increased the respective mRNA expression about twofold without any transcriptional suppression, and the increased expression did not affect the asexual stage development.

### Drug Response of the Transgenic Parasites With Amplification of Drug Resistance-Associated Genes

**Figure 3:**
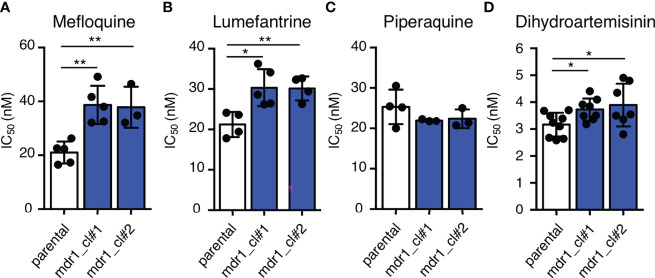
An additional copy of *mdr1* modified susceptibility to multiple antimalarial drugs. **(A–D)** The IC_50_ values were determined for the antimalarial drugs mefloquine, lumefantrine, piperaquine, and dihydroartemisinin with pfcas9-mdr1 parasites (mdr1_cl#1 and mdr1_cl#2) and the pfcas9 parental strain (parental). IC_50_ values of mefloquine **(A)**, lumefantrine **(B)**, and dihydroartemisinin **(D)** with transgenic parasites were higher than those of the parental strain, but the IC_50_ values of piperaquine **(C)** were not significantly different. Mean ± SEM IC_50_ values (**Table 1**) were calculated three to nine times. Statistical evaluations comparing the transgenic parasites and parental strain were performed using Student’s t-tests. *p < 0.05; **p < 0.01.

To examine the effects of an additional copy of *mdr1* on drug susceptibility, we measured the 50% inhibitory concentration (IC_50_) values of pfcas9-mdr1 for the common ACT partner drugs mefloquine, lumefantrine, and piperaquine, and an artemisinin derivative, dihydroartemisinin (**Table 1** and **Figure 3**). The IC_50_ values of mefloquine for two clones of pfcas9-mdr1 were 38.7 and 37.8 nM, which were ~1.8-fold higher than those of parental parasites (IC_50_ value; 21.0 nM; **Figure 3A**). The pfcas9-mdr1 line survived with no morphological changes, even at a mefloquine concentration that killed the parental line (**Supplementary Figure 1**). For lumefantrine, the IC_50_ values of the two clones were 30.3 and 30.1 nM. These values were ~1.4-fold increase compared to the parental line (IC_50_; 21.2 nM; **Figure 3B**). Although the increased expression of *mdr1* mediated by an additional copy insertion confers resistance to mefloquine and lumefantrine, parental and transgenic parasites were not significantly different for piperaquine sensitivity (**Figure 3C**). In addition, the amplification of *mdr1* slightly increased the IC_50_ values for dihydroartemisinin (**Figure 3D**).

**Table 1:** IC_50_ values of mefloquine, piperaquine, lumefantrine, and dihydroartemisinin with pfcas9-mdr1.

Parasite line	Mean ± SEM		Statistical outputs		Mean ± SEM		Statistical outputs	
	MFQ IC_50_ (nM)	N	Notation	p-Value	LMF IC_50_ (nM)	N	Notation	p-Value
Parental	21.0 ± 1.8	5	N.A.	N.A.	21.2 ± 1.6	4	N.A.	N.A.
pfcas9-mdr1 clone#1	38.7 ± 3.2	5	**	0.001	30.3 ± 2.1	5	*	0.012
pfcas9-mdr1 clone#2	37.8 ± 4.4	3	**	0.006	30.2 ± 1.4	4	**	0.006
**Parasite line**	**Mean ± SEM**		**Statistical outputs**		**Mean ± SEM**		**Statistical outputs**	
	**PPQ IC_50_ (nM)**	**N**	**Notation**	**p-Value**	**DHA IC_50_ (nM)**	**N**	**Notation**	**p-Value**
Parental	25.3 ± 3.0	3	N.A.	N.A.	3.17 ± 0.14	9	N.A.	N.A.
pfcas9-mdr1 clone#1	21.9 ± 0.2	3	n.s.	0.325	3.73 ± 0.14	8	*	0.017
pfcas9-mdr1 clone#2	22.4 ± 1.3	3	n.s.	0.421	3.89 ± 0.30	7	*	0.035

Statistical comparisons were made against the parental line. N, number of independent repeats (assays conducted with technical duplicates). Statistics employed unpaired Student’s t-tests.

MFQ, mefloquine; LMF, lumefantrine; PPQ, piperaquine; DHA, dihydroartemisinin; n.s., not significant; N.A., not applicable.

*p < 0.05; **p < 0.01.

**Figure 4:**
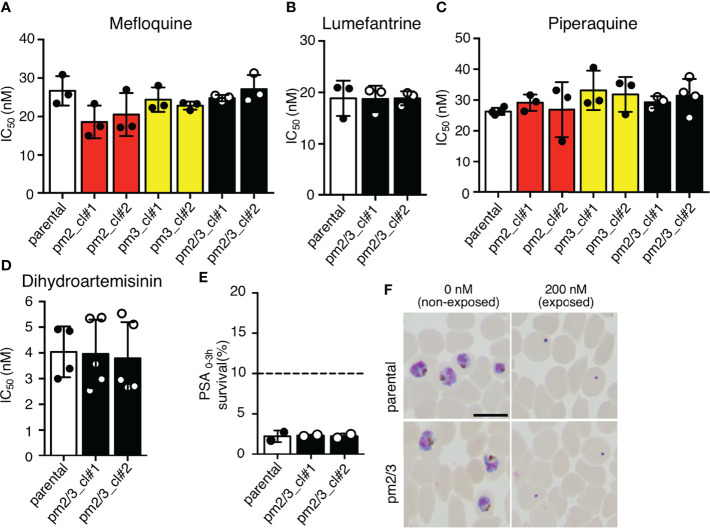
The effect of an additional copy of *pm2* and *pm3* on drug sensitivity. **(A–D)** IC_50_ values were determined for the antimalarial drugs mefloquine, lumefantrine, piperaquine, and dihydroartemisinin with transgenic parasites harboring an additional copy of *pm2* and/or *pm3* (pm2_cl#1, pm2_cl#2, pm3_cl#1, pm3_cl#2, pm2/3_cl#1, and pm2/3_cl#2) versus the pfcas9 parental strain (parental). None of the IC_50_ values for these transgenic parasites were significant versus the parental strain. IC_50_ values (**Table 2**) were calculated three to five times. **(E, F)** *In vitro* piperaquine survival assays (PSA_0–3 h_) were performed for the pfcas9-pm2/3 and the parental strain. **(E)** Survival rates were determined as a percentage of the parasitemia obtained with treated versus drug-off and were not significant comparing transgenic parasites and parental. The dashed line represents the 10% survival rate cutoff that distinguishes piperaquine-resistant (≥10%) from piperaquine-sensitive (<10%) parasites in PSAs. **(F)** Thin blood smears stained with Giemsa reagent were prepared 24 h post-drug withdrawal. pfcas9-pm2/3 was killed by piperaquine treatment, as was the parental strain. N, n = 2 (**Table 2**). A scale bar indicates 10 µm. Significance was determined using Student’s t-tests.

We then analyzed the effects of increased copy numbers of *pm2* and *pm3* on ACT-component drugs by measuring IC_50_ (**Table 2**). Insertion of *pm2* or *pm3* had no effect on the resistance of ACT partner drugs, namely, mefloquine, lumefantrine, and piperaquine, nor on an artemisinin derivative, dihydroartemisinin (**Figures 4A–D**). These results were consistent with a report demonstrating that episomal overexpression of *pm2* or *pm3* did not confer drug resistance (Loesbanluechai et al., 2019). Furthermore, no significant changes were observed in drug resistance by insertion of both *pm2* and *pm3* (**Figures 4A–D**). The amplification of the *pm2* and *pm3* region has been reported to be associated with reduced susceptibility of piperaquine using a PSA (Witkowski et al., 2017). Therefore, we performed a PSA using pfcas9-pm2/3 transgenic parasites and found that an additional copy of both *pm2* and *pm3* had no impact on drug response compared to parental parasites (**Figures 4E, F** and **Table 2**). These results indicate that, at least in the genetic background of the *P. falciparum* reference strain 3D7, a 1.5- to 2-fold increase in *pm2* and *pm3* expression levels is not sufficient to confer detectable drug resistance to piperaquine and other drugs including artemisinin.

**Table 2:** IC_50_ values and PSA for transgenic parasites with increased copy numbers of *pm2* and *pm3*.

Parasite line	Mean ± SEM		Statistical outputs		Mean ± SEM		Statistical outputs	
	MFQ IC_50_ (nM)	N	Notation	p-Value	LMF IC_50_ (nM)	N	Notation	p-Value
parental	26.7 ± 2.2	3	N.A.	N.A.	18.9 ± 2.0	3	N.A.	N.A.
pfcas9-pm2 clone#1	18.6 ± 2.5	3	n.s.	0.071	N.A.			
pfcas9-pm2 clone#2	20.5 ± 3.2	3	n.s.	0.193	N.A.			
pfcas9-pm3 clone#1	24.4 ± 1.8	3	n.s.	0.472	N.A.			
pfcas9-pm3 clone#2	22.8 ± 0.6	3	n.s.	0.169	N.A.			
pfcas9-pm2/3 clone#1	24.8 ± 0.5	3	n.s.	0.454	18.7 ± 1.5	3	n.s.	0.960
pfcas9-pm2/3 clone#2	27.1 ± 2.1	3	n.s.	0.895	18.9 ± 0.8	3	n.s.	1.000
**Parasite line**	**Mean ± SEM**		**Statistical outputs**		**Mean ± SEM**		**Statistical outputs**	
	**PPQ IC_50_ (nM)**	**N**	**Notation**	**p-Value**	**DHA IC_50_ (nM)**	**N**	**Notation**	**p-Value**
parental	26.3 ± 0.6	4	N.A.	N.A.	4.04 ± 0.49	4	N.A.	N.A.
pfcas9-pm2 clone#1	29.2 ± 1.5	3	n.s.	0.105	N.A.			
pfcas9-pm2 clone#2	26.9 ± 5.1	3	n.s.	0.902	N.A.			
pfcas9-pm3 clone#1	33.1 ± 3.7	3	n.s.	0.085	N.A.			
pfcas9-pm3 clone#2	31.8 ± 3.3	3	n.s.	0.109	N.A.			
pfcas9-pm2/3 clone#1	29.3 ± 1.1	3	n.s.	0.052	3.96 ± 0.59	5	n.s.	0.924
pfcas9-pm2/3 clone#2	31.3 ± 2.7	4	n.s.	0.121	3.83 ± 0.65	5	n.s.	0.816
**Parasite line**	**PSA survival rate (%)**	**N**	**Statistical outputs**					
			**Notation**	**p-Value**				
parental	2.23	2	N.A.	N.A.				
pfcas9-pm2/3 clone#1	2.32	2	n.s.	0.877				
pfcas9-pm2/3 clone#2	2.25	2	n.s.	0.973				

Statistical comparisons were made against the parental line. N, number of independent repeats (assays conducted with technical duplicates). Statistics employed unpaired Student’s t-tests.

MFQ, mefloquine; LMF, lumefantrine; PPQ, piperaquine; DHA, dihydroartemisinin; n.s., not significant; N.A., not applicable; PSA, piperaquine survival assay.

### *mdr1* Transcript Levels Correlate With Mefloquine Resistance, But Not Lumefantrine and Dihydroartemisinin

**Figure 5:**
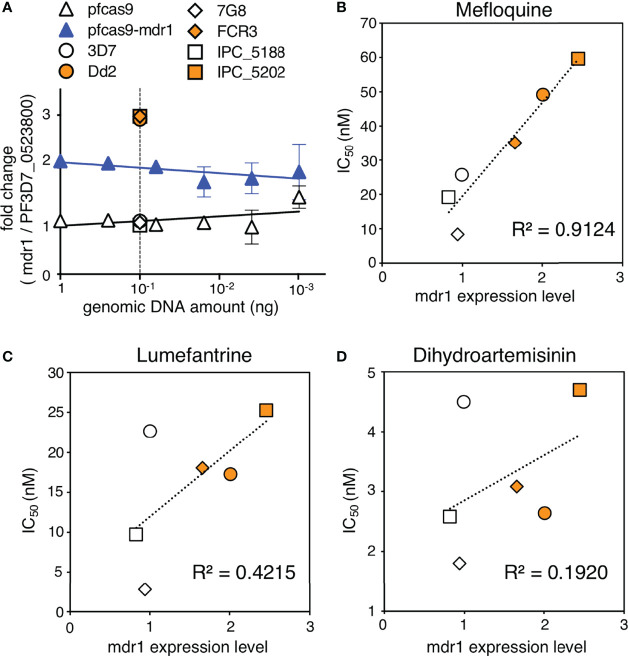
Association of *mdr1* copy number and antimalarial drug sensitivity in laboratory strains. **(A)** The *mdr1* copy number of laboratory strains and transgenic parasites were detected by real-time genomic PCR. Dd2 and FCR3 were used as *mdr1* multicopy reference parasites, and 3D7 and 7G8 were used as *mdr1* single-copy reference parasites. Cambodian field isolates IPC_5188 and IPC_5202 were found to have single and multiple copies, respectively. N, n = 3–4 (**Supplementary Table 3**). **(B–D)** The laboratory strains were assayed for *mdr1* expression levels and IC_50_ values for mefloquine **(B)**, lumefantrine **(C)**, and dihydroartemisinin **(D)**. N, n = 3–9 (**Supplementary Table 3**). There was a linear correlation between the *mdr1* expression levels and mefloquine resistance (R^2^ = 0.91). The association between *mdr1* expression levels and lumefantrine or dihydroartemisinin resistance was unclear (R^2^ = 0.42, 0.19, respectively). Data are shown as mean ± SD. The coefficient of determination, R^2^, was defined using linear regression modeling. The strains with multiple copied *mdr1* are indicated with orange-filled markers.

Copy number variation analysis of patient-derived *P. falciparum* DNA has shown that *mdr1* is associated with antimalarial drug susceptibility (Price et al., 2004; Price et al., 2006; Phompradit et al., 2014; Phyo et al., 2016; Montenegro et al., 2021). We demonstrated that an increase in *mdr1* mRNA expression reduced the susceptibility for mefloquine, lumefantrine, and dihydroartemisinin (**Figure 3**). We next examined whether *mdr1* transcript levels directly correlate with drug resistance using multiple laboratory strains. We used 3D7 and 7G8 as *mdr1* single-copy reference strains and Dd2 and FCR3 lines as *mdr1* multiple copy reference strains (Price et al., 2004; Veiga et al., 2012). The Cambodian IPC_5188 and IPC_5202 isolates, which respectively show susceptible and resistance phenotypes in a ring-survival assay with artemisinin, were used as strains in which the *mdr1* copy number was unknown (Ariey et al., 2014). First, a real-time genomic PCR assay was developed to determine *mdr1* genome copy number by the ddCt method, with PF3D7_0523800 as an internal control gene, which locates on the same chromosome as *mdr1* but outside of the genomic multiplication region of known drug-resistant strains. The results showed that when 3D7, which has one copy of *mdr1*, was used as a control, 7G8 had a single copy of *mdr1*, and Dd2 and FCR3 had multiple copies as reported (**Supplementary Table 3**). The Cambodian isolates IPC_5188 and IPC_5202 were found to contain single copies and multiple copies, respectively (**Figure 5A** and **Supplementary Table 3**). The subsequent quantitative reverse transcription PCR assays revealed that the strains with multi-copy *mdr1* showed higher expression levels than lines with a single copy of *mdr1* (**Figures 5B–D** and **Supplementary Table 3**). We then examined whether there were significant correlations between the *mdr1* expression levels of these laboratory strains and their susceptibility to mefloquine, lumefantrine, or dihydroartemisinin (**Figures 5B–D**). The results indicated that mefloquine resistance is strongly correlated with *mdr1* expression levels (R^2^ = 0.91), while factors other than *mdr1* expression could have larger impacts on resistance to lumefantrine and dihydroartemisinin (**Figures 5C, D**).

## Discussion

Recent studies show that the evolution of *P. falciparum* drug resistance has rapidly compromised the clinical efficacy of ACTs (Ashley et al., 2014; Amato et al., 2017; Witkowski et al., 2017). The design of effective ACT strategies requires an understanding of the local spread of drug-resistant parasites, especially regional resistance to partner drugs. In this report, we determined if duplications of *mdr1*, *pm2*, or *pm3*, which were observed in field-derived drug-resistant parasites, could reduce the susceptibility against ACT partner drugs. By applying the CRISPR/Cas9 genome editing system, we successfully generated transgenic parasites containing additional copies of *mdr1*, *pm2*, *pm3*, or a combination of *pm2* and *pm3*; these gene duplications resulted in approximately twofold increases in target gene transcript levels. We proved by using transgenic parasites that an increased mRNA expression level of *mdr1* directly decreased sensitivity to mefloquine, lumefantrine, and dihydroartemisinin (**Figure 3**). In various genetic backgrounds, the high correlation between the expression level of *mdr1* and the IC_50_ values of mefloquine indicated that the *mdr1* copy number was the most important genetic factor contributing to mefloquine resistance. However, *mdr1* mRNA levels do not correlate with copy number, and this indicates the role of additional aspects that determine *mdr1* expression levels, such as transcription-related factors. Expression levels of *mdr1* were not tightly correlated with sensitivity to lumefantrine and dihydroartemisinin, although insertion of an additional copy of *mdr1* resulted in decreased sensitivities. These results suggest that the sensitivity to those drugs is affected by other factors in the genetic background rather than *mdr1* expression. Our findings are consistent with the epidemiology-based copy number variation analyses of *mdr1*, which reported that mefloquine resistance was strongly associated with *mdr1* copy number (Price et al., 2004; Veiga et al., 2012; Phompradit et al., 2014). The mRNA expression level of *mdr1* should be determined to judge mefloquine-resistant parasites; however, measuring transcript levels is difficult using patient blood due to RNA instability. Therefore, the *mdr1* copy number remains an important marker for surveillance of drug-resistant parasites, and epidemiological investigations should continue.

We also clearly demonstrated that insertion of an additional *pm2* or *pm3* copy into the *P. falciparum* 3D7 strain had no impact on its antimalarial drug sensitivity. These observations agree with the determination that increased expression of *pm2* or *pm3 via* episomal plasmids had no effect on piperaquine susceptibility (Loesbanluechai et al., 2019). The correlation between piperaquine resistance and *pm2/3* amplification has been reported only in Southeast Asia (Huang et al., 2020). Therefore, increasing *pm2/3* expression in the genetic background of strains of Southeast Asian origin may confer piperaquine resistance. In addition, mutations in the chloroquine resistance transporter that differ from mutations conferring chloroquine resistance have been shown to reduce piperaquine sensitivity (Duru et al., 2015; Agrawal et al., 2017; Dhingra et al., 2017; Ross et al., 2018). Also, many parasites with a single copy of *pm2* and *pm3* have been isolated, which have failed dihydroartemisinin/piperaquine combination treatment in epidemiological studies (Huang et al., 2020; Si et al., 2021). Currently, the *pm2/3* copy number variation is being used as a molecular surveillance resistance marker, but further studies are needed to discover the molecular basis of piperaquine resistance and epistatic interactions.

Epidemiology studies have predicted relationships between antimalarial drug resistance and various genetic factors. Genome editing in combination with Cas9-constitutive-expressing parasites and linearized-donor DNA templates enables the knock-in of large-sized DNAs, such as gene coding sequences including regulatory regions (Nishi et al., 2021). This system is useful to evaluate the causality between copy numbers of drug resistance-associated genes and drug resistance. Additional asexually dispensable gene loci are available for the generation of transgenic parasites with higher copy numbers of target genes and subsequent analysis of correlation with drug resistance, of importance since field isolates with three or more copies of specific genomic regions have been found (Ahouidi et al., 2021). Improvement of this system could allow us to find novel associations between gene copy number and antimalarial drug resistance by further copy number variation analysis in field studies. Identifying the relevance of the association through a genome editing-based gene amplification such as used in this study would provide important information for determining drug strategies for malaria control.

## Data Availability Statement

The original contributions presented in the study are included in the article/**Supplementary Material**. Further inquiries can be directed to the corresponding author.

## Ethics Statement

Ethical approvals for the use of human red blood cells (RBCs) and plasma from the Japanese Red Cross Tokyo Blood Center were obtained from the Medical Research Ethical Committee of the Tokyo Medical and Dental University. Written informed consent from the patients/ participants or patients/participants legal guardian/next of kin was not required to participate in this study in accordance with the national legislation and the institutional requirements.

## Author Contributions

RK, SI, and NS conceived and designed experiments. RK and NS conducted experiments and analyzed the data. All authors wrote the manuscript and contributed to the article and approved the submitted version.

## Funding

This work was supported in part by the e-ASIA Joint Research Program (20jm0210061) under Grant number 21wm0225014, supported by the Japan Agency for Medical Research and Development (AMED); Grants-in-Aid for Scientific Research (18K07084 and 19H03459), which was funded by the Japan Society for the Promotion of Science (JSPS); and grants from Mochida Memorial Foundation for Medical and Pharmaceutical Research, SENSHIN Medical Research Foundation and Ohyama Health Foundation. The funders had no role in the study design, data collection, and analysis; decision to publish; or preparation of the manuscript.

## Conflict of Interest

The authors declare that the research was conducted in the absence of any commercial or financial relationships that could be construed as a potential conflict of interest.

## Publisher’s Note

All claims expressed in this article are solely those of the authors and do not necessarily represent those of their affiliated organizations, or those of the publisher, the editors and the reviewers. Any product that may be evaluated in this article, or claim that may be made by its manufacturer, is not guaranteed or endorsed by the publisher.

## Abbreviations

*mdr1*, multidrug resistance protein 1; *pm2*, plasmepsin 2; *pm3*, plasmepsin 3.
