# Supplementary figures and images for: Evaluation of the Effect of Gene Duplication by Genome Editing on Drug Resistance in Plasmodium falciparum
Source: Front Cell Infect Microbiol. 2022 Jul 5;12:915656. doi: 10.3389/fcimb.2022.915656 (PMC9294729; doi:10.3389/fcimb.2022.915656)

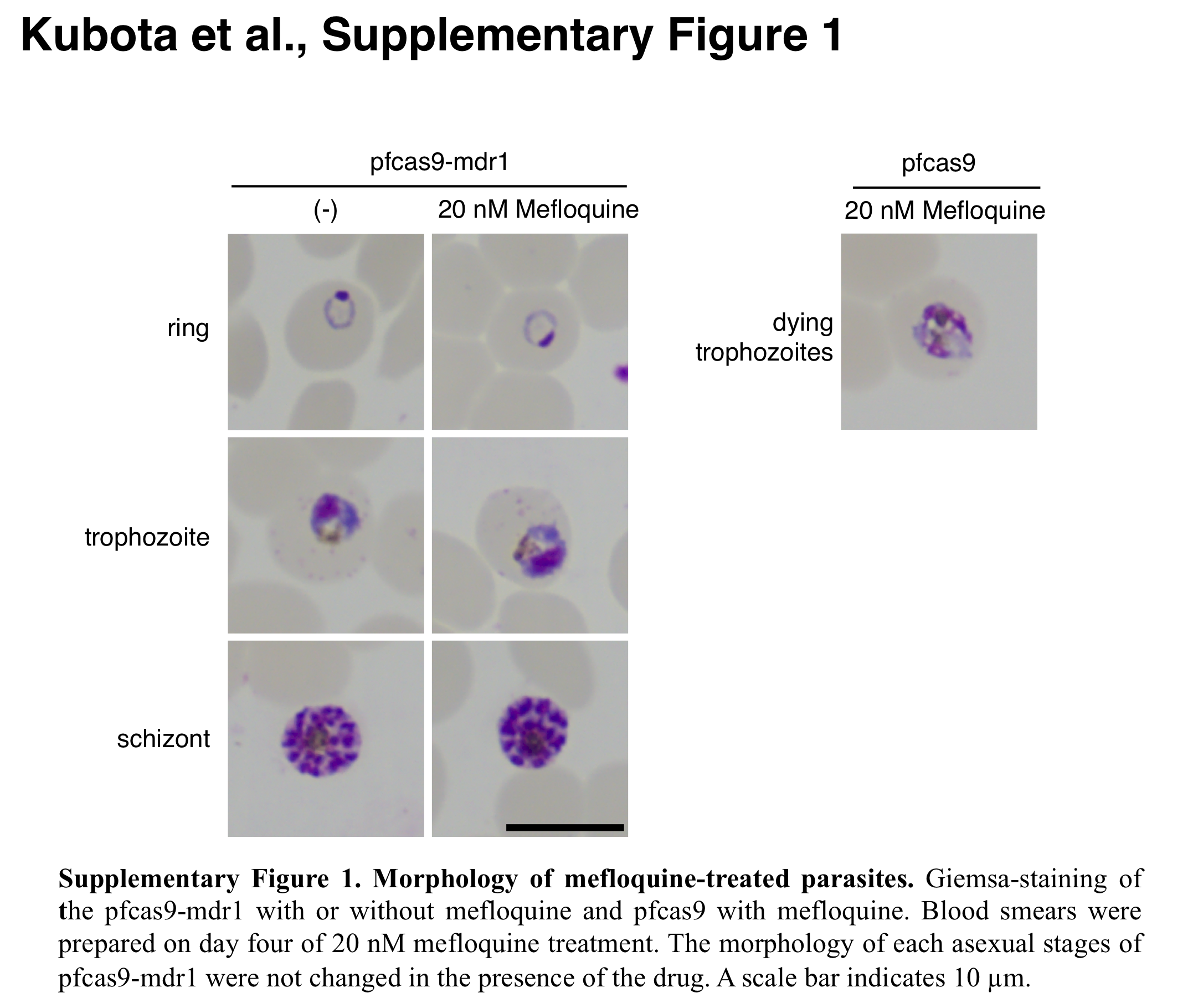

Supplement: Supplementary file 1 [file DataSheet_1.zip › Supplementary Data/Supplementary Figure 1.tif]
